# Oxidative Stress, Parity History, and Remnant Follicles in the Aged Ovary: Insights on Ovarian Cancer Risk and Protection

**DOI:** 10.3390/antiox14070759

**Published:** 2025-06-20

**Authors:** Ulises Urzúa, Arnaldo Marín, Enrique A. Castellón

**Affiliations:** 1 Laboratorio de Genómica Aplicada, Departamento de Oncología Básico Clínica, Facultad de Medicina, Universidad de Chile, Independencia 1027, Santiago 8380453, Chile; 2 Laboratorio de Genómica del Cáncer, Departamento de Oncología Básico Clínica, Facultad de Medicina, Universidad de Chile, Independencia 1027, Santiago 8380453, Chile; arnaldo.marin@uchile.cl; 3 Center for Cancer Prevention and Control (CECAN), Santiago 8331150, Chile; ecastell@uchile.cl; 4 Laboratorio de Oncología Celular y Molecular, Departamento de Oncología Básico Clínica, Facultad de Medicina, Universidad de Chile, Independencia 1027, Santiago 8380453, Chile

**Keywords:** ovarian cancer, ovary, parity, postmenopause, oxidative stress

## Abstract

Ovarian cancer (OC) is the most lethal gynecological cancer globally. Its incidence and mortality consistently rise after menopause. While parity reduces the risk of OC, nulliparity during a woman’s fertile years increases it. Although the association between reproductive history and OC risk is well-established, the long-term impact of pregnancy on the postmenopausal human ovary has received little to no attention. Parity apparently delays the natural decline of the ovarian reserve, but this association also remains unexplored to date. Based on data from cellular, biochemical, and histological markers, as well as epidemiological studies, transcriptomic analyses, and gene knockout mouse models, we review compelling evidence suggesting a critical intraovarian interplay between the residual ovarian reserve and the ovarian surface epithelium (OSE) in the aged ovary. This interaction appears to be a key factor underlying the protective effect of parity on ovarian cancer (OC) risk. We propose that functional FSHR signaling in the remnant follicles of the aged multiparous ovary somehow counteracts the oxidative stress and subsequent chronic inflammation typically observed in the senescent ovary. This mechanism would minimize DNA damage, thereby lowering the probability of neoplastic transformation in the aged mammalian ovary. The precise mechanism by which pregnancy imprints such a long-term follicle–OSE crosstalk warrants further investigation.

## 1. Introduction

Ovarian cancer (OC) is the leading cause of gynecological cancer mortality worldwide [1]. While the precise etiology of OC remains incompletely understood, reproductive history is a well-established factor influencing OC risk [2,3]. Numerous epidemiological studies demonstrate that OC risk increases with uninterrupted ovulation (e.g., nulliparity) and decreases under physiological and pharmacological conditions that temporarily suppress ovulation, such as full-term pregnancies, oral contraceptive use, and breastfeeding [3,4,5]. Furthermore, the incidence and mortality of sporadic OC—that without a hereditary component—steadily rise after menopause, peaking during the post-menopausal period. This age-dependent decline in ovarian function is primarily attributed to a reduced follicular count, which correlates with diminished ovarian synthesis of estrogen and alpha-inhibin. The subsequent disruption of the hypothalamic–pituitary–ovarian (HPO) axis leads to persistently high circulating levels of the pituitary gonadotropins, follicle-stimulating hormone (FSH), and luteinizing hormone (LH). This hormonal shift induces systemic metabolic and inflammatory changes in women and promotes deleterious effects within the ovary, including oxidative stress, fibrosis, senescence, and chronic inflammation [6,7,8]. Crucially, how the OC risk-reducing effect of parity can overcome this multifaceted age-dependent ovarian decline from the fertile to the post-menopausal phase has not been elucidated to date. Moreover, there is a notable absence of data regarding the effects of past parity on molecular pathogenic markers in the human postmenopausal ovary. This review aims to synthesize existing literature to enhance our understanding of the factors promoting early ovarian carcinogenesis during the post-menopausal age, considering the ovary as a primary site of OC origin. The relevance of this topic is further underscored by the increasing trend in developed countries over recent decades for women to remain childless or postpone childbearing [9], which is projected to lead to rising rates of various health issues, including OC, during post-reproductive age.

## 2. Oxidative Stress as a Central Feature of Ovarian Aging

### 2.1. Reactive Oxygen Species in the Fertile-Age Ovary

**Figure 1:**
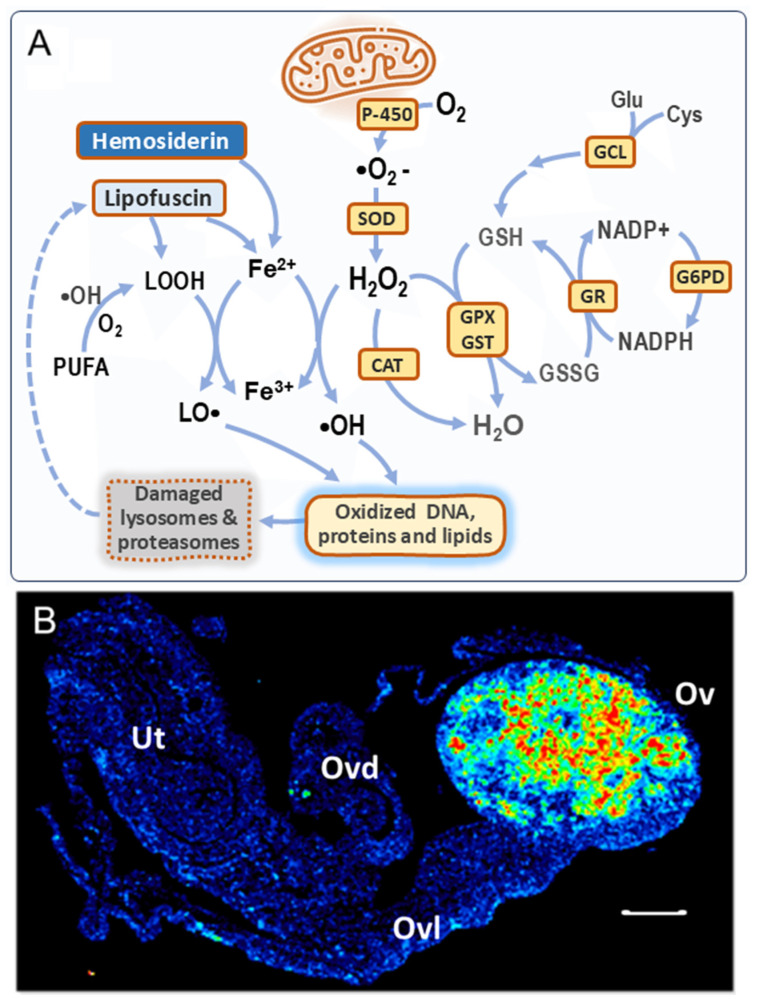
Formation of ROS in the aged ovary. (**A**) Canonical ROS production by mitochondria, the antioxidant system, and additional ROS sources. P-450 = cytochrome oxidases; SOD = superoxide dismutase; CAT = catalase; GPX/GST = glutathione peroxidase/transferase; GR = glutathione reductase; G6PD = glucose-6-P-dehydrogenase; GCL = glutamate–cysteine ligase. In the aged ovary, both lipofuscin and hemosiderin can leak Fe^2+^, which reacts with H_2_O_2_ to produce •OH radicals (Fenton reaction). Lipid peroxides (LOOH) derived from lipofuscin and from polyunsaturated fatty acids (PUFAs) can also react with Fe^2+^ to form LO• radicals [18,19]. Both •OH and LO• radicals cause oxidative damage to biomolecules. Lipofuscin is formed as a consequence of dysfunctional proteasomes and lysosomes due to OS. Further details in the text. (**B**) Lipofuscin imaging in a 16 m.o. nulliparous mouse ovary, detected by autofluorescence; Ov = ovary, Ovd = oviduct, Ovl = ovarian ligament, and Ut = uterine horn; bar = 200 μm. Taken with permission from reference [20]. Lower lipofuscin levels were observed in young [21] and aged parous [20] mouse ovaries.

Processes essential to the reproductive function of the ovary, including ovulation, follicle maturation, atresia, luteinization, and luteolysis, rely on transient but controlled pulses of reactive oxygen species (ROS) [10]. As an inherent aspect of aerobic metabolism, ROS are primarily generated as by-products of the mitochondrial electron transport chain (Figure 1A). The canonical ROS include hydrogen peroxide (H_2_O_2_), its precursor superoxide anion (•O_2_^−^), and the hydroxyl radical (•OH). The •OH is a product of the well-known Fenton reaction between H_2_O_2_ and Fe^2+^ ions. While basal ROS levels are essential for certain cellular functions, an excess of ROS leads to an unbalanced redox state known as oxidative stress (OS), which can inflict damage on cellular components. To counteract this threat, cells possess an antioxidant system comprising enzymes such as catalase (CAT), superoxide dismutase (SOD), and glutathione peroxidase (GPX). Additionally, ROS can be scavenged by the tripeptide glutathione (GSH), which is subsequently oxidized to glutathione disulfide (GSSG). The NADPH-dependent enzyme glutathione disulfide reductase (GSR) can reduce GSSG back to GSH. GSH can also be synthesized de novo from amino acid precursors by the enzymes GSH synthetase (GSS) and glutamate–cysteine ligase (GCL). GCL catalyzes the rate-limiting step of the de novo GSH synthesis. All mentioned ROS and their metabolizing enzymes have been identified in the mammalian ovary [11] (Figure 1A).

Ovulation is a ROS-dependent process that requires the cyclical rupture and subsequent repair of the ovarian surface epithelium (OSE) [12]. This “tear and repair” process can, as a trade-off, induce DNA damage in the OSE [13]. Furthermore, mature follicular fluid contains ROS [11], which can exacerbate oxidative damage to both the OSE and the fimbria upon ovulation. Similarly, follicle atresia, the degeneration of ovarian follicles, is frequently linked to OS across most of the recruited follicular cohort [14]. However, the dominant follicle, which is selected for ovulation, manages to preserve an adequate level of GSH to counteract oxidative damage, thereby allowing its progression towards ovulation [15,16]. Finally, the functional and structural regression of the corpus luteum (CL), a temporary intraovarian endocrine gland, is an apoptosis-mediated process determined by a delicate balance between the activity of antioxidant enzymes and the endogenous ROS generated from its steroidogenic activity [17].

### 2.2. Oxidative Stress in the Aging Ovary Is Linked to Follicle Depletion

Biological aging is characterized as a progressive, time-dependent decline in cell and tissue function. OS is a recognized factor contributing to aging through the oxidative damage of cellular components across virtually all mammalian tissues [22]. In the ovary, this redox imbalance is coupled with the gradual exhaustion of follicles as age advances. This depletion ultimately impacts post-reproductive female physiology at a systemic level, thereby increasing the risk of chronic diseases, including reproductive cancers [23]. This age-dependent OS negatively affects several ovarian cell types, including oocytes, follicular cells (granulosa and theca), stromal cells, the OSE, and resident immune cells [21].

Mitochondrial dysfunction, alongside altered metabolism of glucose, GSH, and phospholipids, contributes to the primary redox imbalance observed in oocytes, follicles, and cumulus cells of aging human ovaries [6]. Similarly, the aging mouse ovary experiences follicle depletion linked to OS, characterized by low expression of both mitochondrial (e.g., peroxiredoxin 3 (Prdx3), thioredoxin 2 (Txn2)) and cytosolic (e.g., glutaredoxin (Glrx), glutathione S-transferase mu 2 (Gstm2)) antioxidant genes, as well as structural damage to ovarian molecular components, including proteins, lipids, and nucleic acids [24].

Lifestyle factors, such as tobacco smoking, a high-fat diet, obesity, and exposure to certain environmental pollutants, endocrine-disrupting agents, and cancer chemotherapy can negatively impact the quantity and quality of follicles, leading to concomitant OS and thus mimicking an early ovarian aging-like phenotype [25,26,27]. These conditions are associated with premature ovarian failure in women and can be modeled in mice through gene silencing or knockdown to identify candidate genes involved in follicle loss associated with redox imbalance. For instance, female mice null for the Gclm gene, which codes for the modifier subunit of GCL, exhibit low oocyte and ovarian GSH levels, resulting in OS and early depletion of ovarian follicles [28,29]. Similarly, female knockout mice for Nrf2 (nuclear factor, erythroid derived 2, like 2) display a low count of primordial follicles at middle age (10–12 months old) [30]. Nrf2 is a transcription factor that regulates the expression of antioxidant genes in many cell types and plays a relevant role during ovarian aging [31].

### 2.3. Oxidative DNA Damage and Disrupted DNA Repair in the Aged Ovary

An excess of ROS can chemically modify the genetic material at both the nucleotide bases and the sugar–phosphate backbone. Specifically, •OH and LO• radicals abstract hydrogen atoms from the bases and the sugar–phosphate backbone of DNA (Figure 1A). The most frequent types of oxidative DNA lesions include base modifications, base loss, single/double DNA strand breaks, protein–DNA adducts, and intra/interstrand DNA crosslinks. The guanine oxidation product, 8-hydroxy-2′-deoxyguanosine (8-OHdG), is widely employed as a marker of oxidative DNA damage [32].

Importantly, two synergistic aspects link cancer and aging concerning oxidative DNA damage: as aging increases OS, the capacity to sense and repair damaged DNA diminishes in aged cells. Specifically, both double-strand break (DSB) repair [33,34] and base-excision repair (BER)—which performs the removal of adducts such as alkylated and oxidized bases—are affected by age-dependent OS [35]. In the ovary, oxidative DNA damage is elevated in stromal ovarian cells of aged mice [20,24]. Furthermore, decreased expression of the DSB repair genes *ATM*, *BRCA1*, *RAD51*, *ERCC2*, and *H2AX* has been observed during follicle and oocyte aging in murine and human ovaries [36,37]. Consistent with these findings, a transcriptome study detected downregulation of over three dozen genes involved in the DNA damage response in aged (16 months old) mouse ovaries compared to young-adult (4 months old) mouse ovaries [38]. Seventeen of these were DSB repair genes, including *Rad51*, *Rad54b*, and *Rad54l*, which function via homologous recombination, as well as the Fanconi anemia complementation genes *Fancd2* and *Brca2*, which colocalize in nuclear foci in response to DNA damage [39].

Interestingly, in the absence of pathogenic mutations directly affecting the homologous recombination repair function of the BRCA1/BRCA2-encoded proteins, the promoter methylation of these genes has been associated with tumorigenesis and poor prognosis in breast/ovarian cancer [40]. In fact, various types of epigenetic alterations have been recently proposed as one of the hallmarks of ovarian aging [41]. Despite the fact that genomic DNA methylation decreases with age, additional epigenetic factors (histone modifications, ncRNAs, or RNA methylation) and upstream transcriptional regulators might modulate this age-concerted downregulation of DNA repair genes. This is an emerging field of research since it links various age-related ovarian pathologies to the epigenetic changes occurring in the ovary that parallel the natural fertility decline in women.

### 2.4. Lipofuscin and Hemosiderin Accumulation Further Contribute to OS in the Aged Ovary

Oxidative DNA damage is one of the major drivers of the aged ovary to a senescent state [8]. Senescence is a cellular response to various stressors leading to irreversible cell cycle arrest, metabolic reprogramming, chromatin remodeling, resistance to cell death, and the production of inflammatory mediators and proteases. Senescence can play divergent roles as a tumor suppressor or tumor promoter [42]. In the early senescent phase, proliferation of cells containing damaged DNA is prevented by induction of p53 and the cyclin-dependent cell cycle kinase inhibitors p21 and p16, among other factors. As this proliferative blockade persists, senescent cells develop a secretory, pro-inflammatory phenotype that modifies the tissue microenvironment, thus promoting endogenous chronic inflammation through increased immune-cell recruitment [8]. Indeed, the transcriptome of the aged mouse ovary shows increased expression of genes involved in inflammatory/immune responses, cell adhesion, TNF synthesis, peptidase activity, wound healing, and immune cell markers [8,21,38]. The ovarian stroma is particularly prone to accumulating senescent cells [8]. In fact, ROS-induced senescent fibroblasts in the mouse ovarian stroma promote neoplastic transformation of c-myc immortalized OSE cells [18].

In addition to modifying DNA, OS can also inflict damage upon various organelles. In organs and tissues characterized by high rates of phagocytosis and autophagy, age-related dysfunction of lysosomes and proteasomes leads to the formation and accumulation of lipofuscin. This intracellular, oxidized, and cross-linked substance is considered a hallmark of senescence [43]. Lipofuscin has been detected in aged murine ovaries [20,21,44,45], specifically inside foamy, enlarged multinucleated macrophages (MNM) associated with stromal cells [44]. Cellular debris resulting from follicular atresia during uninterrupted fertile cycles may contribute to lipofuscin formation as the ovary ages. This phenomenon might explain why the ovary uniquely accumulates lipofuscin within the aged mouse reproductive tract, as depicted in Figure 1B [20].

Furthermore, lipofuscin possesses the ability to trap certain metals, predominantly ionic iron. Interestingly, iron also accumulates with advancing age in several organs in the form of hemosiderin. Hemosiderin is another intracellular, insoluble aggregate containing degraded ferritin plus lysosomal remnants, and it is also found within macrophages [44,46]. Ferritin is a multimeric, non-heme iron carrier and storage protein that predominantly binds ferric ion and is found increased in the serum of post-menopausal women [19]. In situations of iron overload, such as hemolysis after the rupture of blood microvessels (hemorrhage), macrophages engulf erythrocyte contents, mostly hemoglobin, leading to the deposition of hemosiderin [47,48]. Importantly, redox-active iron can be released during heme degradation [49] and from hemosiderin in acidic conditions, such as the lysosome; this also takes place in inflammation and hypoxia [50], both processes exacerbated in aging.

Iron accumulation can induce ferroptosis, an alternative type of cell death characterized by distinct mitochondrial shrinkage, increased ROS, depletion of GSH, and lipid peroxidation without DNA fragmentation or evident chromatin changes [51]. Decreased glutathione peroxidase 4 (GPX4) activity due to low GSH levels is a marker of ferroptosis, and this alteration has been recently described in patients with diminished OR and in human aged ovaries [52]. Relatedly, iron overload and hemosiderin aggregates are observed in the ovaries of an estrogen receptor 1 (*Esr1*)-deficient mouse model along with enlarged MNM, increased mast cells, and dysregulated expression of genes involved in iron transport, storage, and regulation [53]. In fact, the aged mouse ovary overexpresses the iron ion transporters NRAMP (*Slc11a1*) and ferroportin (*Slc40a1*) [38], the latter an exporter of ferrous iron to the plasma, which has been implicated in iron overload diseases [54]. As shown in Figure 1A, non-heme free ferrous ion (Fe^2+^) reacts with H_2_O_2_ to form •OH radicals. In turn, OH• reacts with polyunsaturated fatty acids (PUFAs) to form lipid peroxides (LOOH), which can also react with Fe^2+^ to form LO• radicals. Both •OH and LO• are able to induce oxidative damage to macromolecules and organelles. Therefore, as lipofuscin and hemosiderin apparently colocalize in the MNM of the aged mouse ovary [20], this might represent an additional source of redox-active iron that amplifies ROS levels in senescent cells [55], in addition to those produced by dysfunctional mitochondria [6]. As mentioned above, the OS perpetuated by such mechanisms ends up depleting GSH levels, thus resulting in iron-mediated damage, ferroptosis, and chronic inflammation [8].

## 3. Aging and Reproductive History Modulate Ovarian Cancer Risk

### 3.1. Ovarian Cancer Risk Increases at Menopause and Decreases by Prior Parity

Coincident with the age-dependent increase in OS and the diminished DNA repair capacity of the mammalian ovary, OC mortality and incidence steadily rise as women approach menopause—the hallmark of reproductive aging—which typically occurs at a mean age of 51 years [2]. As previously mentioned, the primary underlying cause of reproductive aging is the depletion of ovarian follicles, leading to decreased circulating levels of steroid hormones and a concomitant increase in the pituitary gonadotropins, FSH and LH, due to the disruption of the HPO axis [2].

Despite significant follicle depletion at menopause, the postmenopausal ovarian stroma retains a partial steroidogenic capacity [56,57,58]. Testosterone, androstenedione, and estradiol [59], as well as the receptors for estrogen (ER-alpha), androgen [60], and the enzymes 17-beta hydroxysteroid dehydrogenase type-1 (HSD17B1) and aromatase (CYP19A1), have been detected in various cell types of the postmenopausal ovary [61,62]. Consistent with increased circulating gonadotropin levels, their cognate receptors, FSHR and LHR, are expressed in human postmenopausal ovaries [63] and in aged female mice [21,38].

In addition to age, a woman’s reproductive history during her fertile life significantly influences her risk of developing OC later in the postmenopausal period. Specifically, periods that suppress ovulatory cycles, such as pregnancy, oral contraceptive use, and breastfeeding, are consistently linked to a significantly decreased OC risk [3,4,5]. Regarding parity, a meta-analysis of 32 studies reported a relative risk of 0.46 for women with three or more births compared to nulliparous women [5]. Notably, the reduction in OC risk conferred by pregnancy and oral contraception is directly proportional to the number of full-term births and years of use, respectively. Furthermore, the magnitude of this decreased risk is similar for both parity and oral contraceptive use [3,4,5].

This reduced OC risk is linked to the transient suppression of ovulatory cycles and supports the “incessant ovulation” theory as an etiological factor in ovarian carcinogenesis [2]. As discussed, the ROS-dependent, repetitive tear-repair damage to the OSE, coupled with the exposure of the OSE and fimbria to ROS-containing follicular fluid during each ovulatory event, is believed to increase the likelihood of DNA damage [12,13] that may remain unrepaired as aging progresses [20,36,37].

Conversely, limited attention has been paid to the long-term effects of pregnancy, oral contraceptive hormones, and breastfeeding in preventing ovarian carcinogenesis beyond simply pausing ovulation. High progesterone levels during pregnancy have been proposed to preclude the mitogenic action of low-to-moderate estrogen levels, thereby reducing the chances of malignant transformation [64]. However, precisely how this ovarian exposure to progesterone during fertile life confers a long-term reduced OC risk at menopause remains largely unknown.

Parity also reduces the risks of endometrial and breast cancers. A recent follow-up population study demonstrated a linear reduction in the incidence of ovarian, endometrial, and breast cancers for each additional childbirth across the entire fertile age range studied (<20 to 45 years old). This reduction occurred at similar rates for all three cancers, suggesting a possible common underlying biology [65]. However, while the link between parity and breast cancer risk has been explored in postmenopausal breast tissue, even identifying specific genes and pathways [66,67,68], analogous studies associating parity with OC through gene expression analyses of postmenopausal ovarian tissue are exceedingly scarce. Thus, the evidence reviewed here aims to address this knowledge gap by exploring how parity modulates long-term gene expression in the postmenopausal ovary.

### 3.2. Parity Delays the Natural Decline of the Ovarian Reserve

Age-dependent follicle depletion is accurately expressed as the decrease in the ovarian reserve (OR), a term defining the total number of primordial (non-growing) follicles that contain the entire pool of oocytes potentially fertilizable during the reproductive life of females. In mammals, the OR is typically established in utero around birth and declines steadily during early adult life, accelerating with advanced reproductive aging [69]. In women, this decline accelerates during their mid-to-late 30s. Over the first decade of life, all primordial follicles remain quiescent due to inhibitory mechanisms within both the oocytes and the pre-granulosa cells [70]. At menarche, a cohort of primordial follicles, containing oocytes in meiotic arrest, are activated upon the release of these inhibitory signals [71]. Once activated, typically only a single follicle develops and matures towards ovulation, while the remaining ones undergo degradation through atresia. From menarche to menopause, this process repeats periodically during fertile life, leading to progressive follicle depletion. The initial OR consists of approximately half a million to one million primordial follicles, yet only around 500 are ovulated during a woman’s fertile lifespan, and nearly 1000 primordial follicles remain in the human ovary at menopause [72].

As described previously, certain conditions and exposures have been associated with an accelerated rate of OR decline, mirroring the effects of aging [25,26,27]. Experimental evidence suggests that accelerated OR depletion results from the recurrent activation of dormant follicles, which leads to increased ovarian OS, early ovarian aging, and an augmented risk of postmenopausal pathologies [70,71]. Conversely, other interventions and agents that delay the OR depletion can attenuate ovarian aging and thereby promote healthy reproductive aging in females. These include caloric restriction; certain hormones, such as melatonin, ghrelin, and anti-Müllerian hormone (AMH); and energy-metabolism regulating compounds, such as metformin, rapamycin, and nicotinamide mononucleotide [27,73,74].

Emerging epidemiological evidence indicates that parity may be another factor delaying OR decline. A large cross-sectional analysis, encompassing 10 cohorts with over 3800 women aged 21–57 years, found significantly higher circulating levels of AMH—an established OR marker—in parous versus nulliparous women, particularly in those aged 40 and above [75]. Another cross-sectional study, which aimed to analyze both reproductive and lifestyle AMH determinants in 2320 women with a mean age of 37.3 (±9.2) years, found a positive association between higher parity and higher age-specific AMH levels [76]. Lastly, a smaller study involving 186 women aged 20–35 years observed higher plasma levels of AMH in multiparous compared to nulliparous women. Ovarian volume and antral follicle number—additional OR markers—were also elevated in the parous condition [77].

**Figure 2:**
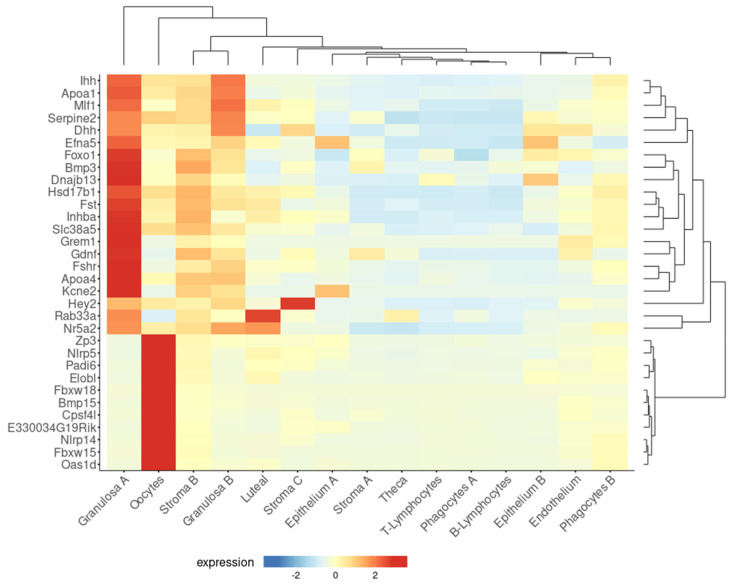
A subset of genes upregulated in the aged multiparous ovary is predominantly expressed by granulosa cells and oocytes. The Shiny tool (https://omrf.shinyapps.io/ovarianagingscatlas/, last accessed on 24 January 2025) contains single-cell RNA-seq data for the 15 distinct cell types identified in the middle-aged mouse ovary [21] and was employed to predict which specific cell types predominantly express each of the 32 follicle/oocyte-related genes that were upregulated in the aged multiparous relative to the aged nulliparous mouse ovary [38]. Gene symbols were entered into the “bubbleplot-heatmap” tool and grouped by cluster names. Plot type settings were as follows: heatmap, scale gene expression, cluster genes, and cluster samples to visualize dendrograms in both axes.

Consistent with the above epidemiological data, a higher residual follicle count was detected in multiparous compared to nulliparous mouse ovaries, both 21 months old (equivalent to post-menopause) [38]. Moreover, microarray analysis showed higher expression of 32 genes involved in follicle and oocyte homeostasis in the ovaries of aged multiparous mice compared to the aged nulliparous condition. Using a tool designed to explore single-cell RNA-seq data of a recent study on mouse ovarian aging [21], this 32-gene set was confirmed to be highly expressed in two primary types of granulosa cells (A, B) and oocytes and at lower levels in stroma B cells (Figure 2). Diverse granulosa cell types are constituents of follicles at different stages of development, including preantral, antral, mitotic, and atretic [21]. Twenty-one out of the thirty-two genes were expressed by granulosa cells A. In turn, from these 21, 11 showed predominant expression in mitotic, 3 in atretic, and 3 in antral granulosa cells [21]. The genes *Fshr*, *Inhba*, *Foxo1*, *Nr5a2*, *Gdnf*, and *Efna5* are involved in hormone response, while the oocyte-expressed genes *Nlrp5*, *Nlrp14*, *Padi6*, *Bmp15*, *Zp3*, and *Oas1d* are the target genes of transcription factors involved in the maintenance and survival of primordial follicles [78], reflecting a follicle–oocyte interdependence. Given that parity seems to delay the natural OR decline, it sounds conceivable to attribute the reducing OC risk effect to a higher residual OR persisting in the mammalian parous ovary at post-menopausal age. The functionality of this residual OR in the senescent, post-reproductive ovary has not been characterized so far.

To date, there is little information on how pregnancy might slow the natural OR decline. As with the presumed effects of transient ovulatory suppression [3,4,5] or high progesterone levels in decreasing OC risk [64], these pregnancy factors could somehow promote a long-term intraovarian homeostasis of follicles and oocytes. Clues may arise from the processes of CL formation, maintenance, and regression during pregnancy. The CL is considered a transient intraovarian “gland” originating from the remaining theca and mural granulosa cells of the mature Graafian follicle that differentiate into progesterone-producing luteal cells [79]. Limited amounts of progesterone are produced by granulosa cells during follicular development, but excess progesterone can inhibit follicular development at the primordial–primary transition [80]. During pregnancy, high progesterone levels derived from the CL could diffuse inside the ovary to inhibit the maturation of primordial follicles. Compared to the non-parous condition, this may result in a higher AMH level, i.e., a higher OR if measured postpartum, since serum AMH tends to decrease during gestation [81]. Upon delivery, the progesterone drop would allow resuming follicle development. A long-term epigenetic effect of progesterone on the OR cannot be ruled out, but this has not been experimentally addressed so far.

As described above, the association of the OR size with age and reproductive history comes from cross-sectional studies. To date, no longitudinal study has addressed the OR–parity association. Instead, some metabolic parameters and the exposure to certain environmental agents have been analyzed with this approach. A few examples include the association of AMH with serum lipid levels as a risk factor of cardiovascular disease, which was studied during 12 years of follow-up in a cohort of 1015 Iranian women [82]. In a recent study, the association of air pollutant exposure with longitudinal AMH levels was studied for over 20 years in 2574 women from the Netherlands [83]. Another study addressed the association of serum AMH levels with markers of bone density and turnover during ∼14 years of follow-up [84]. Future longitudinal studies in the above range of follow-up periods are expected to confirm the OR–parity association.

## 4. Follicle–OSE Interactions and FSHR Signaling

### 4.1. A Paracrine Crosstalk Between Follicles and the Ovarian Surface Epithelium

As discussed previously, the decline in the reproductive function of the ovary, specifically the depletion of germ cells and follicles, is intrinsically linked to an increased risk of ovarian cancer (OC). An additional characteristic of the aging process in the mammalian ovary is the proliferation of epithelial structures, such as clefts and inclusion cysts, which form from invaginations of the ovarian surface epithelium (OSE) into the ovarian stroma [85,86]. Compared to the OSE—which is actually a mixed or uncommitted epithelium—these cysts are enriched in epithelial markers, suggesting that a mesenchymal-to-epithelial transition likely led to their formation [87]. Importantly, clefts and inclusion cysts have been implicated in metaplasia and neoplastic transformation, thus being considered precursor lesions of OC [88].

Similar to the effects of aging, the ovaries of mice with genetically induced loss of germ cells and follicles typically develop OSE hyperplasia that invades the stroma, leading to the formation of tubular adenomas [89]. For instance, in *Rev7* mutant mice, the complete loss of oocytes and follicles was concomitant with increased gonadotropin levels, ovarian DNA damage, and the development of tubulostromal adenomas [90]. Another illustrative case is the white spotting variant (Wv) mouse, which carries a spontaneous *c-kit* mutation resulting in early follicular loss. In fact, Wv ovaries exhibited an inverse relationship between the levels of alpha-inhibin (a granulosa cell marker) and cytokeratin-8 (an epithelial cell marker). Interestingly, alpha-inhibin was detected in stromal remnants of degenerated follicles, and even in a *TP53* knockout background, a minimal number of ovarian follicles were able to suppress OSE hyperplasia in this model [91]. Further studies have suggested that the depletion of germ cells and follicles creates an ovarian environment conducive to the oncogenic transformation of OSE cells [92,93]. This inverse relationship between follicle content and OSE proliferation supports the existence of a growth-inhibiting paracrine factor produced by follicular granulosa cells [93]. This factor presumably diminishes or becomes absent upon follicular depletion due to age (or other aforementioned factors), thereby promoting epithelial proliferation.

**Figure 3:**
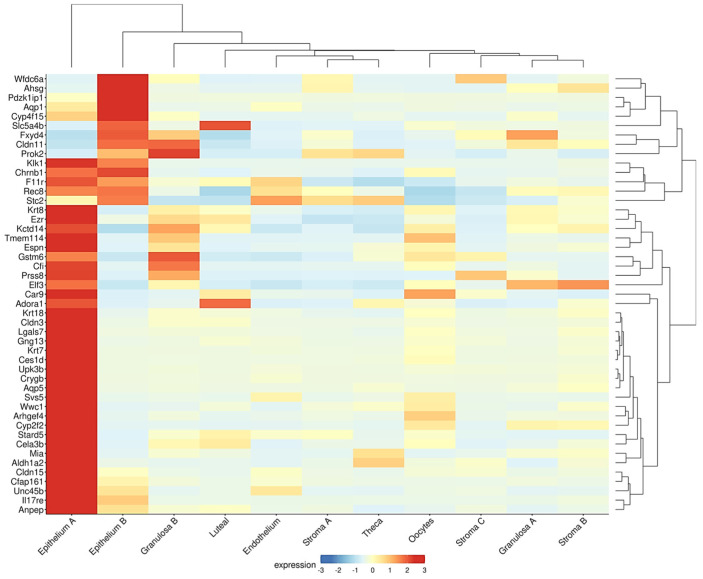
Genes upregulated in the aged nulliparous mouse ovary are mostly of epithelial origin. As described in legend to Figure 2, the Shiny tool was used to track the expression of the 112 genes up-regulated in the aged nulliparous mouse ovary [96] across the 15 cell types identified in the middle-aged mouse ovary [21]. A total of 47 out of the 112 genes showed predominant expression in “epithelium A” and “epithelium B” cell types. Immune cells were omitted from the heatmap. Display parameters were those described in Figure 2.

A similar inverse association between the epithelial and follicle compartments was observed in the ovaries of the Fshr knockout (FORKO) mouse. This model exhibits a decreased OR and an early menopause-like phenotype, accompanied by OSE thickening, cytokeratin-positive stromal cysts, high expression of tight-junction proteins, and serous papillary cystadenomas [94,95]. Interestingly, a gene signature resembling that of the FORKO model was detected in a recent transcriptome analysis of the aged nulliparous mouse ovary [96]. In this reproductive condition, *Fshr* was downregulated with a concomitant overexpression of epithelial genes, such as the cytokeratins *Krt7*, *Krt8*, *Krt18*, and *Krt23*, and the cell junction genes *Cldn3*, *Cldn11*, *Cldn15*, *F11r*, and *Ezr* [96]. Moreover, when displayed in a cell-type context (Figure 3), a total of 47 genes upregulated in the aged nulliparous mouse ovary showed predominant expression in the epithelium-A and epithelium-B cell types [21], representing 42% (47/112) of all the genes upregulated in the nulliparous, high OC risk condition [96]. The cytokeratin and cell junction protein-coding genes, in addition to another gene subset including *Rec8*, *Aldh1a2*, *Espn*, *Gng13*, *Cfi*, *Stard5*, and *Crygb*, were also found coordinately dysregulated in the pre-neoplastic phase of the spontaneous transformation of cultured mouse OSE cells in a previous transcriptome study [97]. In summary, the inverse association between the sizes of the follicular and the OSE-derived epithelial components observed in the FORKO mouse ovary [94,95] also appears to occur in ovaries with divergent parity histories [38,96], thereby providing insight into the mechanistic bases of the protective effect of pregnancy on OC risk.

Similar to the *Fshr* knockout mice, additional gene expression signatures from the mouse knockouts of *Pten*, *Smad3*, and *Cdh1* were also significantly enriched in the transcriptomic profile of the aged nulliparous mouse ovary [96]. These genes are also linked to follicle homeostasis and have recognized roles as tumor suppressors. The downregulation of *Pten* in oocytes provokes a massive activation of follicles, resulting in OR depletion [70], whereas the transcription factor *Smad3*, a downstream member of TGF-beta signaling, has been found to inhibit mouse OSE proliferation [98].

### 4.2. FSHR Expression in the Aged Ovary: Does It Hold the Gonadotropin Theory of Ovarian Cancer?

Expressed by the granulosa cells, the *Fshr* gene codes for the FSH receptor, a G-protein-coupled receptor that plays a pivotal role in female reproductive function. In response to FSH, FSHR promotes follicular development and mediates estrogen synthesis in the ovaries during fertile age [99]. FSHR displays four alternative splicing isoforms, where FSHR1 acts through the canonical Gαs/cAMP/PKA pathway, and FSHR3 exhibits growth factor activity via MAPK–ERK signaling [100]. Consistent with age-dependent follicle depletion, *Fshr* expression decreases with age in human and mouse ovaries [21,38] but can still be detected in granulosa cells and other ovarian cell types, such as the OSE [63]. According to a single-cell RNA-seq analysis, *Fshr* expression in the middle-aged mouse ovary is predominantly follicular [21], i.e., highest in granulosa A cells, significantly lower in granulosa B and stroma B cells, and negligible in the epithelial cell types (Figure 2). The role of this low but detectable *Fshr* expression in the post-reproductive ovary remains largely unknown.

As described, follicular *Fshr* expression restricts OSE hyperplasia, an observation linking parity history to a higher ovarian reserve (OR) and reduced OC risk. It therefore seems plausible to consider a protective role of *Fshr* expression in residual follicles of the aged parous ovary resulting from former pregnancies [38]. However, this idea might appear to contradict the “gonadotropin theory” of OC, which is based on the well-known increase of FSH and LH in circulation at menopause and the growth-promoting, migratory, and invasive effects of these gonadotropins on OC cells [101]. Such cancer-promoting actions are mediated by FSH signaling through the FSHR3 variant, which is predominantly expressed by the fertile-age OSE and in OC cells [100].

Nevertheless, both earlier and recent evidence challenge the gonadotropin theory. Firstly, FSH is subject to age-dependent post-translational modifications in the FSHβ subunit that modulate its action. In younger, ovulating women, the partially/hypo-glycosylated FSH21/18 form predominates, while the hyper-glycosylated FSH24 form increases with aging and is predominant in peri- and post-menopause [102]. The binding of FSH24 to the FSHR delays the dissociation of FSHR oligomers into monomers [103] and reduces the proliferative response of follicles [104]. Besides this age-altered action of FSH on granulosa cells, a study with OSE cells from healthy donors reported a growth inhibitory effect of FSH in postmenopausal OSE samples [105]. Additionally, gonadotropins, including FSH, have been used as ovulation-inducing drugs in fertility treatments, but their possible effects on OC risk are controversial. Earlier studies claimed an increased OC risk, but a recent meta-analysis indicated no increased risk of invasive OC regardless of fertility drug use. A specific increased risk of borderline OC was found only in nulliparous women [106]. Another literature review concluded that mostly observational studies have suggested a link between fertility drugs and various cancers, but a significant association is lacking due to limitations in sample sizes, adequate follow-up, and uncontrolled confounding factors in those studies [107].

Secondly, epidemiological analyses linking endogenous FSH levels and OC risk have provided conflicting results. While some reports suggest that high FSH levels are significantly associated with increased OC risk in pre- and post-menopausal women [108], other studies fail to confirm a direct correlation between FSH levels and OC risk. Among the latter, a nested case-control study of 88 patients with primary invasive epithelial OC compared to 168 matched, cancer-free control women showed that FSH levels were not significantly different between women who were subsequently diagnosed with OC and control women. Moreover, when FSH levels were ranked in tertiles (low, medium, high), women in the highest tertile did not have a higher OC risk relative to the lowest tertile [109]. Another nested case-control study in 67 epithelial OC patients showed that high prediagnostic FSH levels were associated with lower OC risk. In this case, FSH levels were ranked in tertiles (low, medium, or high) according to the distribution of FSH levels among control women [110]. A recent analysis in 370 premenopausal women indicated that higher FSH was directly associated with an increased risk of type-II OC but inversely associated with type-I OC [111].

Part of the reason for conflicting data regarding the role of the FSH/FSHR axis on OC risk can be context-dependent. While hormones such as FSH can regulate repair, differentiation, and stress responses to maintain the homeostasis of normal ovarian tissue, in the context of OC and other endocrine-related malignancies, FSH can play a role as a tumor promoter by influencing OC onset, progression, and metastasis. As mentioned above, this OC promoter action of FSH can be mediated by the alternative FSHR3 variant [100]. In addition, FSHR has been proposed as a therapeutic target [112], but, again, this contradicts a recent report indicating that Fshr expression seems to act as a good prognostic factor in OC [113]. Moreover, high FSHR expression in tumor cells and tumor vasculature was associated with a longer progression-free survival and overall survival. Conversely, low FSHR protein expression was associated with shorter survival parameters in high-grade OC, while blockage of FSHR expression increased the invasive ability of OC cell lines OVCAR-3 cells and COV362 [113]. The latter findings might have therapeutic applications. Small molecules such as Org41841, a type of thienopyr(im)idine that increases human FSHR expression in the plasma membrane [114], could be tested as a therapeutic co-adjuvant in OC patients. Such approaches would be worth attempting in the future.

### 4.3. A Proposed Role for FSHR Signaling in Protection Against OC in the Aged Parous Ovary

Fifteen years ago, Huhtaniemi conducted a critical analysis of the gonadotropin effect on carcinogenesis. His primary conclusion was that the “gonadotropin theory” was predominantly based on in vitro studies and that “the clinical and experimental evidence in vivo was fragmentary, weak, and controversial” [115]. Much of the data reviewed here from various sources confirm that a convincing and definitive link between FSH and increased ovarian cancer (OC) risk is still lacking. In contrast, a significant body of novel evidence discussed herein suggests that FSHR expression in the aged ovary is dependent on a higher residual ovarian reserve (OR) resulting from past pregnancies compared to the aged nulliparous condition. Importantly, this higher FSHR expression in parous women could account for their observed lower OC risk.

Then how could these remnant follicles contribute to protecting the aged ovary? Two possible, non-exclusive scenarios can be proposed. The first was introduced in Section 4.1 above: the granulosa cells (and oocytes) from residual follicles could secrete a paracrine factor that diffuses inside the ovarian stroma to suppress the OSE proliferation of normal, pre-neoplastic, or inclusion cysts [93]. Inhibin-alpha and TGF-beta have been proposed as candidates for this role [91,92,93], and a recent work shows that AMH secreted by ovarian follicles inhibits the proliferation of mouse OSE cells [116]. These proteins belong to the TGF-beta/BMP superfamily, a class of growth factors implicated in the formation, assembly, and activation of primordial follicles [117]. In coherence with this idea, the TGF-beta/BMP family members *Inhba*, *Bmp3*, *Fst*, and *Grem1* were among the overexpressed genes in the aged multiparous mouse ovary [38]. *Inhba* codes for the inhibin subunit beta A, a monomer of the dimeric protein complexes activin-A, activin-AB, and inhibin-A. As their names suggest, the systemic action of these dimers is to either activate or inhibit pituitary FSH secretion, respectively. During aging and menopause, circulating inhibin-A (inhibin-alpha) levels decrease along with estrogens [118], while activin-A increases parallel to the FSH rise [119]. In human and mouse ovaries of fertile age, activins promote the assembly of primordial follicles, thereby influencing the size of the primordial follicle pool [120]. Thus, the concomitant expression of the TGF-beta/BMP antagonists, follistatin *(Fst*) and gremlin-1 (*Grem1*), would be part of a mechanism to counteract the growth-promoting effect of activins, a role proposed in human postmenopausal ovaries [58]. Besides the mentioned TGF-beta/BMP members, the genes *Ihh*, *Hey2*, *Efna5*, *Serpine2*, and *Zp3* were also overexpressed in the aged multiparous mouse ovary compared to its nulliparous counterpart [38]. According to the Gene Ontology (GO) database, these genes are also involved in growth regulation.

The ovarian FSH/FSHR axis offers a second potential explanation for the role of remnant follicles in reducing ovarian cancer (OC) risk. We hypothesize that FSHR signaling plays a protective role against OS in the parous ovary in a manner similar to how it promotes the survival of growing follicles during folliculogenesis. In this process, most of the recruited follicles undergo atresia due to OS, with the exception of the dominant follicle, which maintains a sufficient GSH level to reach the antral state. This antioxidant protection is dependent on FSH/FSHR signaling in granulosa cells [11,15,16]. Importantly, as androgens predominate in the aged ovary [56,58,59] and prevent ovulation, such an active FSH/FSHR axis in the parous condition would maintain granulosa cells of residual follicles in a quiescent state with an adequate GSH level. In fact, the postmenopausal ovary has been regarded as an androgen-producing and gonadotropin-driven organ [121], a notion implying functional FSH/FSHR signaling and supported by the observation that androgens like dehydroepiandrosterone enhance follicular viability and responsiveness in an animal model of menopause [122]. Thus, a key aspect of this hypothetical scenario is how GSH levels are sustained by the remnant follicles of the aged parous ovary to counteract age-dependent OS. In this regard, an interesting question would be to evaluate if the menopause-predominant hyperglycosylated FSH, which has a reduced proliferative effect [104], is still capable of maintaining a sufficient GSH level. High expression and activity of GCL in follicular granulosa cells determines increased synthesis of GSH [123]; therefore, GCL activity could serve as a readout of FSH functionality and GSH levels.

Further clues supporting the link between parity history and GSH levels come from a recent study suggesting elevated expression of the glutathione transferases *Gstm6* and *Gsta3* and glutathione peroxidase *Gpx3* in the aged nulliparous mouse ovary [96]. This represents a high-risk OC condition with reduced Fshr expression, analogous to the FORKO model. GSH acts as a substrate for GPX and GST enzymes, which detoxify H_2_O_2_ and xenobiotic compounds (Figure 1A). Assuming a positive correlation between transcript and GPX/GST enzyme levels, excessive H_2_O_2_ and lipid hydroperoxides (LOOH) would lead to GSH depletion in the aged nulliparous condition. Consequently, low GSH levels would result in a reduced GSH/GSSG ratio, a typical marker of OS and an additional readout of FSHR functionality. Other redox-related genes overexpressed in the aged nulliparous ovary were alcohol dehydrogenase (*Adh7)* and the aldehyde dehydrogenases *Aldh1a2* and *Aldh1a7* [96], which detoxify reactive aldehydes derived from lipid peroxides [124] (i.e., LOOH), as also depicted in Figure 1A. Finally, a behavioral model of ovariectomized (menopause-like) Fshr knockout mice (Fshr−/−) resulted in severe OS with a low GSH/GSSG ratio in the whole brain of these animals [125]. Although a different organ and disease context, this finding resembles and supports the hypothesized mechanism that we propose to occur in the parous ovary.

## 5. Concluding Remarks and Perspectives

This review compiles information from diverse sources regarding reproductive history as a major OC risk factor and the biological processes underlying this effect. Multiple lines of evidence are presented in the context of age-related ovarian decline and the potential protective action of parity. The text emphasizes the complex interactions among OS, aging, and reproductive history in modulating OC risk. Though full-term pregnancies and nulliparity significantly influence the risk, the precise causes of OC remain unclear. The transition from fertile age to menopause involves significant hormonal changes, notably in the gonadotropins FSH and LH. These shifts impact ovarian function and are associated with systemic metabolic and inflammatory changes that contribute to both ovarian aging and increased cancer susceptibility.

OS is a key factor in normal ovarian function, with ROS playing a central role in processes such as ovulation, follicle maturation, and luteal regression. While basal levels of ROS are necessary for certain cellular functions, their overproduction leads to OS and subsequent DNA damage, thereby increasing the risk of OC. In the aging ovary, OS is directly related to the OR decline, the follicle depletion, and the increase of both reproductive aging and cancer susceptibility. Environmental factors, such as smoking, obesity, and exposure to toxicants, can further exacerbate OS, promoting ovarian aging and, in some cases, leading to premature ovarian failure. Aged ovarian cells exhibit impaired DNA repair mechanisms, increasing susceptibility to mutations and genomic instability, which are hallmarks of carcinogenesis. Senescence in the aged ovary indicates a cellular state that could either inhibit or promote tumorigenesis, with the precise role of senescence in cancer initiation remaining context-dependent.

**Figure 4:**
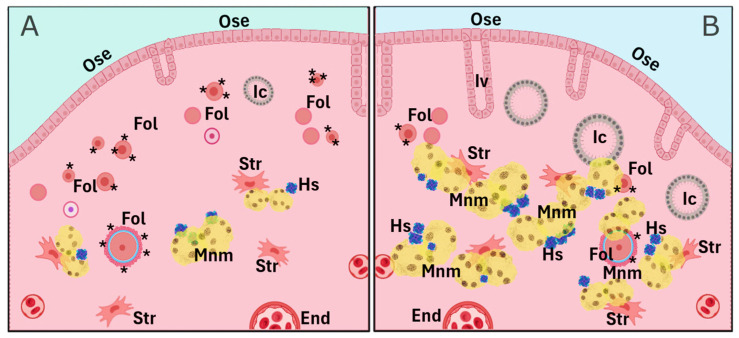
Comparative scheme of parity-related cell and tissue markers depicting the long-term effect of parity on the aged ovary. The parous condition (**A**) contains a higher number of remnant follicles (Fol) and lower counts of lipofuscin-containing multinucleated macrophages (Mnm), hemosiderin (Hs) aggregates, epithelial invaginations (Iv), and inclusion cysts (Ic), while the nulliparous condition (**B**) shows a lower follicle count and increased numbers of Iv, Ic, Mnm, and Hs. Further cell types and structures include the ovarian surface epithelium (Ose), stromal cells (Str), and endothelial cells (End). Asterisks in follicles depict the FSH/FSHR axis. Figure was created using a generic template from BioRender (www.biorender.com, last accessed 3 February 2025).

As previously mentioned, cumulative evidence suggests that pregnancy, oral contraceptive use, and breastfeeding—conditions that temporarily suppress ovulation—are significantly associated with a reduced risk of OC later in menopause. Parity, specifically, reduces OC risk proportionally to the number of full-term pregnancies. This protective effect is likely attributable to the hormonal changes during pregnancy, particularly elevated progesterone levels, and their influence on the reproductive tract, especially the ovary. A less explored effect is the influence of parity on delaying the natural decay of the OR, meaning the preservation of a greater number of primordial follicles. Indeed, women who have given birth tend to have higher AMH levels, a well-established OR marker, particularly in women over 40 years of age. The missing, but key, link between ovarian progesterone exposure and the long-term improved follicle homeostasis is largely unknown and requires further research. In animal models, parity is linked to a higher number of residual follicles in the aging ovary, with gene expression profiles suggesting preserved follicle and oocyte homeostasis (Figure 2). These residual follicles may confer protection against OC by preventing OSE proliferation via paracrine signaling. On the other hand, the aged nulliparous ovary displays increased epithelial structures (Figure 3 and Figure 4) and multiple signs of cell stress beyond those induced by aging. Between these divergent risk conditions, we propose that FSHR expression in the aged parous ovary might play a critical role in maintaining follicle viability and regulating OS, thereby reducing OC risk. A scheme compiling all the above-described evidence and proposed hypotheses is shown in Figure 4.

In summary, further research is needed to better understand the molecular mechanisms underlying the effects of parity, hormonal changes, inflammation, and FSHR signaling in aging ovaries. Such knowledge could contribute to designing preventive strategies and therapeutic interventions for OC. Given the decreasing fertility trends coupled with increased life expectancy in developed countries, this research becomes particularly relevant as increasing rates of postmenopausal health issues, including OC, would be expected.

## Data Availability

No new data was created. All data presented is available from their respective sources, including links and references.

## Abbreviations

The following abbreviations are used in this manuscript:

AMH	Anti-Mullerian hormone
BER	Base excision repair
CAT	Catalase
CL	Corpus luteum
DSB	Double-strand break
FORKO	FSHR knockout
FSH	Follicle-stimulating hormone
FSHR	Follicle-stimulating hormone receptor
GLC	Glutamate–cysteine ligase educed glutathione
GPX	Glutathione peroxidase
GSH	Reduced glutathione
GSR	Glutathione disulphide reductase
GSS	Glutathione synthetase
GSSG	Glutathione disulphide
GST	Glutathione transferase
HPO	Hypothalamic pituitary ovarian
LH	Luteinizing hormone
LHR	Luteinizing hormone receptor
LOOH	Lipid peroxide
MAPK-ERK	Mitogen-activated protein kinase–extracellular signal-regulated kinase
MNM	Multinucleated macrophage
OC	Ovarian cancer
OR	Ovarian reserve
OS	Oxidative stress
OSE	Ovarian surface epithelium
PUFA	Polyunsaturated fatty acid
PFS	Progression-free survival
ROS	Reactive oxygen species
SOD	Superoxide dismutase
TGF-beta/BMP	Transforming growth factor beta/bone morphogenetic protein
Wv	White spotting variant
